# CC chemokine receptor 10 cell surface presentation in melanocytes is regulated by the novel interaction partner S100A10

**DOI:** 10.1038/srep22649

**Published:** 2016-03-04

**Authors:** F. Hessner, C. P. Dlugos, T. Chehab, C. Schaefer, B. Homey, V. Gerke, T. Weide, H. Pavenstädt, U. Rescher

**Affiliations:** 1 Institute of Medical Biochemistry, Center for Molecular Biology of Inflammation, and Interdisciplinary Clinical Research Centre, University of Muenster, Von-Esmarch-Str. 56, D-48149 Muenster, Germany; 2 Department of Internal Medicine D, Molecular Nephrology, University Hospital of Muenster, Albert-Schweitzer Campus 1, A14, and Interdisciplinary Clinical Research Centre, D-48149 Muenster, Germany; 3 Department of Dermatology, Heinrich-Heine-University Düsseldorf, Universitätsstr. 1, D-40225 Düsseldorf, Germany

## Abstract

The superfamily of G-protein-coupled receptors (GPCR) conveys signals in response to various endogenous and exogenous stimuli. Consequently, GPCRs are the most important drug targets. CCR10, the receptor for the chemokines CCL27/CTACK and CCL28/MEC, belongs to the chemokine receptor subfamily of GPCRs and is thought to function in immune responses and tumour progression. However, there is only limited information on the intracellular regulation of CCR10. We find that S100A10, a member of the S100 family of Ca^2+^ binding proteins, binds directly to the C-terminal cytoplasmic tail of CCR10 and that this interaction regulates the CCR10 cell surface presentation. This identifies S100A10 as a novel interaction partner and regulator of CCR10 that might serve as a target for therapeutic intervention.

Dysregulated G-protein-coupled receptor (GPCR) signal transmission is found in a plethora of pathophysiological scenarios^1^ and is reflected by the fact that GPCRs are the most important drug targets^2^. Upon ligand binding, GPCRs signal via activation of heterotrimeric G-proteins leading to the regulation of multiple downstream effectors. Protein interactions with the cytosolic part(s) of GPCRs add an additional layer of regulation to GPCR mediated cellular responses, and the impact of an exhaustive number of GPCR interacting proteins that modulate GPCR signal transduction is now widely acknowledged^3^. The concept of GPCR signalling has been further extended to include G-protein independent signalling pathways mediated via arrestins that act as scaffolds for large signalling complexes^4^. CCR10 (previously known as orphan GPR-2) was identified as the specific receptor for the chemokine CCL27/CTACK^5^,^6^ which is selectively expressed in skin keratinocytes^7^. It belongs to the chemokine receptor subfamily of GPCRs and in addition to CCL27 has CCL28/MEC as a known ligand^8^. CCR10 is expressed in many melanoma cell lines and in cytokine-stimulated melanocytes and CCL27-triggered CCR10 activation has been shown to positively affect immune evasion of melanoma cells^5^,^9^. CCR10 is also expressed on memory T-cells, where it functions in T-cell homing to inflamed skin^10^,^11^. The finding that CCL28 is expressed in epithelial cells of mucosal tissue, such as the gut and lung^12^,^13^, might link CCR10 activation to colonic inflammation^14^. CCR10 is also expressed in several other cell types such as myeloid and endothelial cells where it is upregulated together with its ligand CCL28 in rheumatoid arthritis^15^ arguing for a more general function of ligand-activated CCR10 in immune responses and tumour progression. However, there is only limited information on the intracellular regulation and transport of CCR10.

This report identifies S100A10 as novel interaction partner and regulator of CCR10. This small, dimeric EF hand containing protein of the S100 protein family forms a heterotetrameric complex with annexin A2 (anxA2) and is known to regulate several plasma membrane resident channels and receptors by affecting their trafficking, plasma membrane stability and possibly also activity^16^. We show that S100A10 binds directly to the C-terminal cytoplasmic tail of CCR10 and that this interaction regulates the CCR10 cell surface presentation. This identifies S100A10 as a CCR10 interacting protein that modulates CCR10 availability and might serve as a target for therapeutic intervention.

## Results

### S100A10 regulates CCR10 cell surface presentation

**Figure 1:**
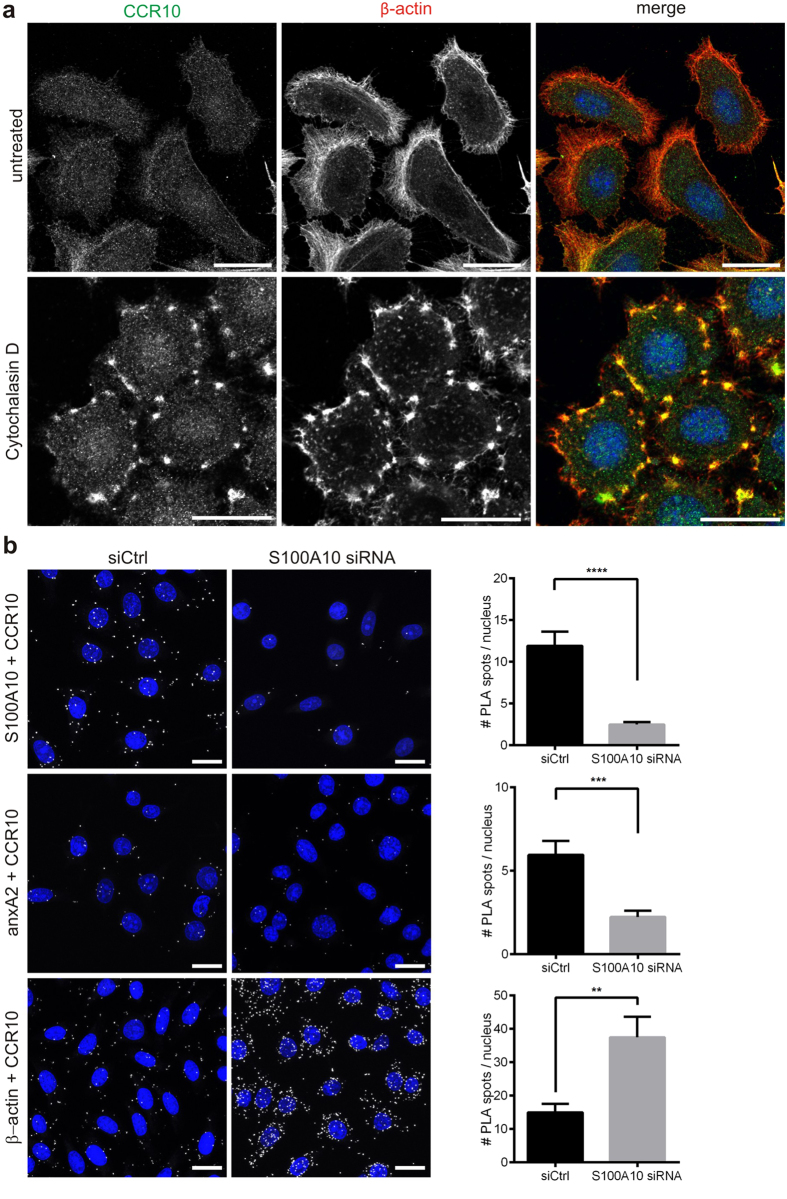
CCR10 interacts with S100A10, anxA2 and β-actin in melanoma cells. (**a**) Confocal images of CCR10 and β-actin distribution in UKRV-Mel-4 cells treated or not with 1 μM cytochalasin D for 30 min. DRAQ5 was used for nuclear staining. Scale bars represent 20 μm. (**b**) *In situ* proximity ligation assays for CCR10 interactions with the anxA2-S100A10 subunits and actin were performed in S100A10 depleted or control (siCtrl) cells. Scale bars represent 20 μm. White spots indicate interaction. Nuclei were stained with DAPI to visualise cells and PLA spots per cell were determined and are given as means ± s.e.m. (please notice the difference in y-axis). For quantitative analysis of PLA, means were calculated from 236–363 cells per condition, and the significance of the observed differences was tested via two-sided unpaired *t*-test. A *p*-value of <0.05 was considered significant. **p < 0.01; ***p < 0.005; ****p < 0.0001.

We first visualised the distribution of CCR10 in the human melanoma cell line UKRV-Mel-4 known to express CCR10^17^ using confocal immunofluorescence microscopy. As shown in Fig. 1a, CCR10 was mostly detected at the cell boundaries, where it colocalised with cortical actin. Interestingly, when the cells were treated with cytochalasin D, a fungal toxin that inhibits actin polymerization and thereby causes actin filament disruption, CCR10 gave strong signals in the remaining actin patches at the plasma membrane (Fig. 1a), suggesting that CCR10 might interact with actin or actin-associated proteins. Because the heterotetrameric complex consisting of anxA2, a member of the Ca^2+^ and phospholipid binding annexin protein family^18^, together with S100A10, a member of the S100 family, associates with cortical actin^19^, and because the S100A10 subunit of the complex interacts with a number of plasma membrane resident channels and receptors^16^, we wondered whether the anxA2-S100A10 complex might have a role in directing CCR10 to actin-rich membrane regions. To specifically target the anxA2-S100A10 complex, we chose to interfere with S100A10 expression. We first established efficient knockdown of S100A10 using S100A10 specific siRNA and non-targeting siRNA as control. Quantitative analysis of western blots confirmed that the levels of S100A10 were effectively reduced, while anxA2 and the unrelated gene product tubulin were not affected. Importantly, CCR10 expression levels remained unchanged in S100A10 ablated cells (Supplementary Fig. S1). To study the interaction of CCR10 with actin and the anxA2-S100A10 complex, we employed the *in situ* proximity ligation assay (PLA) technology. PLA signals only develop when the two bound antibodies are in close proximity (i.e. when their targets interact). No signals were observed in the negative control experiments without primary antibodies (Supplementary Fig. S2). Bright spots appeared when anti-CCR10 antibodies were combined with antibodies against either actin or the anxA2-S100A10 complex, as shown in Fig. 1b. Quantitative analysis confirmed the close association of CCR10 with actin and also revealed an association with the anxA2-S100A10 complex within UKRV-Mel-4 cells. Ablation of S100A10 did not only reduce PLA signal levels for CCR10/S100A10, but also for CCR10/anxA2, suggesting that CCR10 is physically linked to S100A10 within the anxA2-S100A10 complex, probably giving rise to a ternary complex. Surprisingly, quantitative analysis of the PLA signals for CCR10 and actin in S100A10-depleted cells revealed a significant increase as compared with control cells, strongly arguing for a role of the anxA2-S100A10 complex in regulating the CCR10-actin interaction and thereby also CCR10 trafficking.

**Figure 2:**
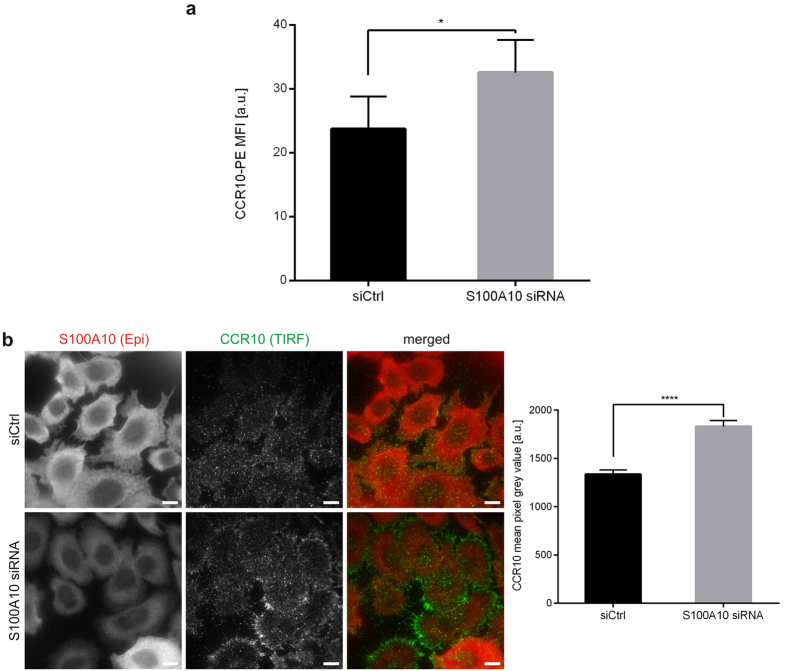
CCR10 surface presentation is increased in S100A10 depleted melanoma cells. (**a**) Flow cytometric analysis of CCR10 levels on the surface of UKRV-Mel-4 cells transfected with non-targeting control siRNA (siCtrl) or S100A10 specific siRNA. Bars represent mean geometric MFIs ± s.e.m. of CCR10-PE antibody signal [a.u. = arbitrary unit]. (**b**) UKRV-Mel-4 cells transfected with control or S100A10 specific siRNA were stained with anti-S100A10 (red) and anti-CCR10 (green) antibodies and analysed via TIRF microscopy. Epi, epifluorescence. Scale bars = 10 μm. Bars represent CCR10 mean pixel grey values ± s.e.m. [a.u. = arbitrary unit]. For flow cytometric comparison of CCR10 cell surface presentation, MFI obtained from 5 independent transfection experiments (with at least 10,000 events per condition of each transfection) were analysed by two-sided paired *t*-test. For TIRF analysis, CCR10 mean pixel grey values in the evanescent field obtained from 144 cells transfected with non-targeting control siRNA and 162 cells transfected with S100A10 specific siRNA were calculated and the significance of the difference was evaluated using two-sided unpaired *t*-test. A *p*-value of <0.05 was considered significant. *p < 0.05, ****p < 0.0001.

To directly address the link between anxA2-S100A10 and CCR10 trafficking, we used flow cytometry to measure the amount of CCR10 at the cell surface. As shown in Fig. 2a, the pool of CCR10 presented at the cell surface of S100A10 ablated cells was significantly increased when compared to control cells transfected with non-targeting siRNA. To confirm this, we selectively visualized the plasma-membrane associated pool of CCR10 by total internal reflection fluorescence (TIRF) microscopy. As shown in Fig. 2b, CCR10 at the cell borders stained more prominently in cells that were depleted of S100A10 (identified by epifluorescence imaging mode) than in control cells, and quantitative analysis of the data confirmed that CCR10 cell surface pools increased significantly upon S100A10 ablation. No apparent colocalisation of the plasma membrane-associated pool of CCR10 with S100A10 was detected in cells transfected with control siRNA (Supplementary Fig. S3), supporting the above observations that S100A10 negatively affects CCR10 trafficking to or stabilisation at the actin-rich cell cortex.

### CCR10 interacts with the anxA2-S100A10 complex via direct binding to S100A10

**Figure 3:**
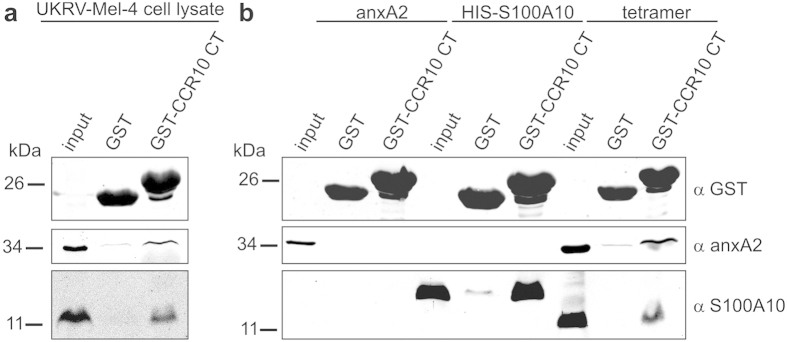
CCR10 interacts with the S100A10-anxA2 tetrameric complex via S100A10. GST-CCR10 fusion protein containing the C-terminal tail (CT) of human CCR10 and GST (as a negative control) were immobilized on Glutathione sepharose beads and then incubated with (**a**) malignant melanoma cell line UKRV-Mel-4 lysates or (**b**) purified recombinant human anxA2, recombinant HIS-tagged human S100A10, or native anxA2-S100A10 tetramer isolated from porcine intestinal mucosa. Interacting proteins were analysed by immunoblotting with the indicated antibodies. Full uncropped scans of blots are shown in Supplementary Fig. S5.

To study the interaction of the anxA2-S100A10 complex with CCR10 more precisely, we used the GST pull-down technique. The cytosolic C-terminal tail of human CCR10 was cloned and expressed as a GST fusion protein (CCR10 CT_314–369_). Purified GST-CCR10 CT was bound to glutathione sepharose beads and then incubated with whole cell homogenates of UKRV-Mel-4 cells. In accordance with our PLA data, the anxA2-S100A10 heterotetramer was readily detected by western blotting of proteins eluted from GST-CCR10 beads (Fig. 3a). Eluates from control beads loaded with GST showed only faint background signals, arguing that the interaction of the two proteins with the CCR10 cytosolic tail was specific. AnxA2 and S100A10 form a highly symmetrical heterotetrameric complex consisting of a S100A10 dimer and two anxA2 monomers^16^. To determine whether association with the CCR10 tail could be attributed to either S100A10 or anxA2, or required the preformed complex, we performed pull-down assays with recombinantly expressed and purified S100A10, anxA2, and native heterotetramer purified from porcine intestinal mucosa. We observed that anxA2 did not interact with the C-terminal domain of CCR10, whereas S100A10 and the heterotetrameric complex did (Fig. 3b). These results revealed that binding of CCR10 to the anxA2-S100A10 tetramer was direct and specific and showed that the interaction was mediated via physical interaction with the S100A10 subunit and did not require the presence of anxA2.

### Mapping of the S100A10 binding site in CCR10

**Figure 4:**
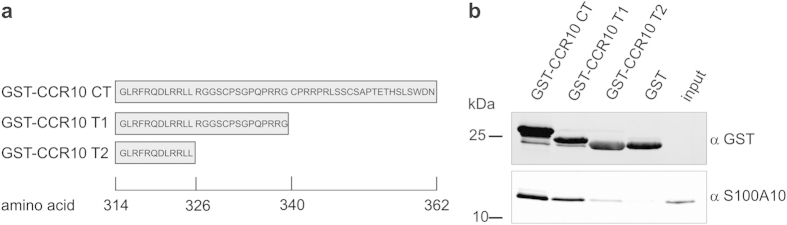
Mapping of the S100A10 binding site in the CCR10 C-terminal tail. (**a**) Linear representation of the C-terminus of CCR10 fused to GST, and the two truncation constructs T1 and T2. (**b**) Binding of GST-CCR10 CT, T1 and T2 to HIS-tagged S100A10 was analysed by pull-down and immunoblotting of the glutathione sepharose-bound proteins with the antibodies indicated. Blots are representative of at least five experiments. Full uncropped scans of blots are shown in Supplementary Fig. S5.

To map the domain necessary for interaction with S100A10, we expressed deletion mutants of the C-terminal tail of CCR10 (Fig. 4a) and compared their ability to bind to S100A10 (Fig. 4b). The T1 mutant could still interact with S100A10, whereas the binding was almost abolished when CCR10 was further truncated (T2 mutant), suggesting that the S100A10 binding site is located within amino acid residues 326 to 339 of the CCR10 C-terminal region.

### The CCR10 binding site in S100A10 is different from the anxA2 binding site

**Figure 5:**
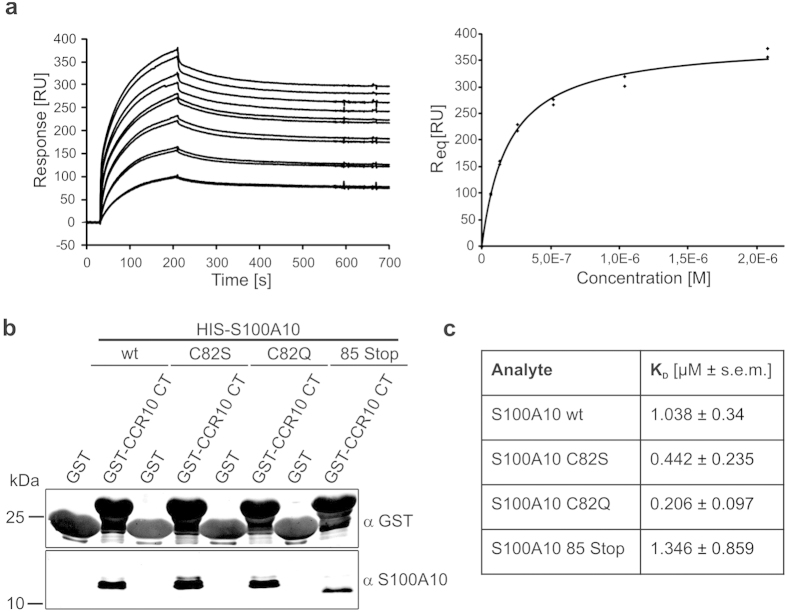
The anxA2 binding motif in S100A10 is dispensable for CCR10 interaction. (**a**) Titration series and kinetics of wild-type HIS-S100A10 binding to immobilized GST-CCR10 CT were determined using surface plasmon resonance (SPR). RU = resonance units, R_eq_ = response at equilibrium. (**b**) Binding of HIS-tagged S100A10 wt and the mutants C82S, C82Q and 85 Stop to GST-CCR10CT was analysed by pull-down and immunoblotting of the glutathione sepharose-bound proteins with the antibodies indicated. Full uncropped scans of blots are shown in Supplementary Fig. S5. (**c**) Comparison of the *K*_D_ values ± s.e.m. as determined by SPR. Each titration series was repeated multiple times leading to at least six data points per S100A10 derivative and concentration.

To determine the affinity of the S100A10-CCR10 interaction, using surface plasmon resonance (SPR), S100A10 at various concentrations was added to GST-CCR10 immobilized on the SPR sensor chip. Analysis of the binding data obtained (Fig. 5a) revealed an affinity of ~1 μM. The highly affine binding of S100A10 and anxA2 within the heterotetrameric complex is structurally well understood^20^. The S100A10 binding site in anxA2 is found in the first 14 N-terminal amino acid residues which organize into an amphipathic α-helix that inserts into one of the two anxA2 binding pockets of the S100A10 dimer. However, we could not detect any resemblance of the putative S100A10 binding region in the CCR10 tail to the anxA2 N-terminal region or to other S100A10 interacting motifs, suggesting that CCR10 might interact with a distinct binding site in S100A10. Therefore, we next asked whether CCR10 could bind to S100A10 mutants showing either severely impaired (C82S) or totally abolished (C82Q, 85Stop) anxA2 binding. As shown in Fig. 5b, wild-type S100A10 and the anxA2 binding-deficient S100A10 mutants co-pelleted in the GST pull-down assays to a similar extent. Analysis of the respective *K*_D_ values revealed that the mutants bound with comparable or even higher affinities to the CCR10 tail (Fig. 5c, and Supplementary Fig. S4). These results together with the fact that S100A10 is able to physically interact with CCR10 and anxA2 at the same time, strongly argue for the presence of an independent binding site for CCR10 in the S100A10 molecule which enables the formation of a tripartite higher order complex consisting of CCR10, S100A10 and anxA2.

## Discussion

Because heterologous overexpression of GPCRs in cell lines might fail to reflect the GPCR activation pattern seen in the native cellular environment, we analysed CCR10 localisation in the malignant melanoma UKRV-Mel-4 cell line which expresses a substantial amount of endogenous CCR10^17^. Interestingly, our data indicate that CCR10 seems to closely associate with cortical actin in these cells. The importance of precise tempo-spatial control of actin reorganisation for directional chemotaxis is widely acknowledged, yet components of the signalling pathway(s) from chemokine-activated GPCRs to the actin cytoskeleton only start to emerge^21^. Evidence for a direct GPCR/actin interaction is still lacking, however, several actin-associated proteins have been reported to simultaneously bind to GPCRs and actin^22^,^23^,^24^ and this might alter the receptor mobility and promote clustering of the receptors and components of the signalling network at the leading edge. Our results show that CCR10 is found in close proximity to cortical actin, although the interaction might be through other actin-associated proteins, as shown for example for the D2 dopamine receptor^22^,^25^, and the exact nature of the CCR10-actin association remains to be determined. We observed increased signals for the CCR10/actin association in S100A10 depleted cells and this was accompanied by increased levels of CCR10 cell surface presentation, suggesting that S100A10 is a modulator of CCR10 cell surface presentation that probably depends on interactions with the actin cytoskeleton.

S100A10 belongs to the S100 proteins that bind Ca^2+^ via two EF hand binding motifs and form dimeric complexes. Conformational changes upon Ca^2+^ binding enable the dimers to interact with a broad range of target proteins, thereby transferring the Ca^2+^ signal to cellular pathways. Interestingly, S100A10 is Ca^2+^ -insensitive due to substitutions in both EF hands, and three-dimensional structural analysis revealed that the Ca^2+^ -free S100A10 adopts a conformation similar to that of other Ca^2+^ -bound S100 proteins, resulting in permanent activation and thus Ca^2+^ -independent binding to cellular targets^20^. Together with anxA2, a member of the annexin protein family of Ca^2+^ -binding proteins that frequently interact with S100 proteins, S100A10 forms heterotetramers and these heterotetramers have been found to regulate the trafficking and/or plasma membrane expression of a number of ion channels and cell surface receptors. The anxA2-S100A10 complex positively affects the surface presentation and/or activity of the TRPV5 and 6 Ca^2+^ channels, the sodium channel Na(V)1.8, the acid sensing ion channel ASIC1a and the serotonin receptor 5-HT1B^26^,^27^,^28^,^29^. We report here a negative correlation between S100A10 expression levels and cell surface presentation of CCR10. Notably, a similar inverse relationship was observed between S100A10 and the potassium channel TASK-1^30^. In the latter example, efficient forward trafficking of S100A10-bound TASK-1 was proposed to be impaired by a transplantable ER retention motif detected at the C-terminal end of S100A10. In line with the suggested role of S100A10 as a cytosolic retention factor, increased cell surface presentation of TASK-1 was observed upon S100A10 knockdown or co-expression of S100A10 mutants without a functional ER retention motif.

How could the changes in CCR10 levels at the cell surface observed in S100A10 downregulated cells be brought about? We found that S100A10 interacts physically with the carboxy-terminal cytosolic tail with the S100A10 binding site situated within amino acid residues 326–340 of CCR10. Consensus sequence analysis yielded no similarities to the S100A10 interaction motif (XOOXXOOX, O = hydrophobic, X = any amino acid residue) in the anxA2 N terminus, and most importantly, S100A10 mutants with severely impaired or even abolished ability to bind anxA2 still bound to CCR10. These findings are in line with the observation that anxA2 did not directly interact with CCR10, but was found associated with CCR10 and S100A10, suggesting that S100A10 bears distinct binding sites for anxA2 and CCR10, and that the S100A10 dimer might act as a linker between CCR10 and anxA2 within a tripartite complex. GPCR activation typically affects the second messenger-dependent protein kinases PKA and PKC, both of which are supposed to regulate the anxA2-S100A10 interaction through antagonistic impact on phosphorylation of a critical Ser residue in the anxA2 N–terminal S100 binding site^16^,^31^,^32^,^33^,^34^. Experimental evidence points to a positive correlation between PKA activation and the formation of the anxA2-S100A10 complex via PKA-mediated phosphatase activity on the anxA2 N-terminal part^32^,^33^,^34^. GPCRs such as CCR10 that couple to Gα_i_ and inhibit adenylate cyclase^35^, might therefore favour anxA2-S100A10 complex disassembly. As the above mentioned ER retention motif is situated in a C-terminal region in S100A10 which is required for high affinity anxA2 binding^16^, this site is most likely covered when S100A10 is complexed to anxA2. Based on these assumptions, we propose that activated CCR10/G_i_-mediated decrease in cAMP levels would favour dissociation of anxA2 from the tripartite complex. The concomitant increase in S100A10 with the ER retention motif now exposed might keep newly synthesized CCR10 away from the cell surface. Subsequent CCR10 signal termination and rising cAMP levels would promote anxA2 binding and mask the ER retention motif, allowing anterograde forward traffic. Thus, regulated formation of a tripartite complex might function in stop-and–go CCR10 forward traffic, thus establishing a negative feedback loop and oscillatory waves of CCR10 activity.

## Methods

### DNA constructs

For expression of the GST-CCR10 fusion proteins, the cDNA encoding the C-terminal tail of human CCR10 (amino acid residues 314–362) was inserted into the pGex-4T-1 GST expression vector. To generate the CCR10 tail truncation mutants T1 (amino acid residues 314–339) and T2 (amino acid residues 314–325), stop codons were inserted into pGex-CCR10CT_314–362_ by site directed mutagenesis. Human S100A10 and S100A10 mutants tagged on the N-terminus with poly-histidine (HIS) were constructed by inserting the respective cDNAs into the pET28a(+) vector. The plasmid encoding human anxA2 was previously described^36^. Details on cloning and mutagenesis procedures are available from HP. All plasmids were verified by sequencing.

### Protein expression and purification

Expression of wild type and mutant CCR10 constructs in *E. coli* BL21 (DE3)pLysS was induced with 0.5 mM isopropyl-β-D-thiogalactoside (IPTG) for 4 h at 30 °C. Cells were pelleted, resuspended in PBS supplemented with protease inhibitor cocktail, and lysed by passing through a French press (1,000 psi) twice. High speed centrifugation (25,000 x g for 30 min at 4 °C) supernatants were incubated with glutathione sepharose for 90 min at 4 °C. Subsequently, beads were washed with PBS containing 0.1% Triton-X 100 and 300 mM NaCl and used for interaction studies.

Expression of poly-histidine (HIS)-tagged human wild type and mutant S100A10 in *E. coli* BL21(DE3)pLysS was induced with 1 mM IPTG at 37 °C for 3.5 h. Cells were pelleted and were resuspended in lysis buffer (50 mM Tris-HCl, pH 7.5, 300 mM NaCl, 20 mM imidazole, 1 mM EDTA, supplemented with 10 mM β-mercaptoethanol and Roche complete protease inhibitor cocktail), and lysed by passing twice through a French press (1,000 psi). Supernatants obtained after high speed centrifugation (100,000 × g, 60 min, 4 °C) were incubated with Ni-NTA agarose for 90 min at 4 °C. After extensive washing in lysis buffer, proteins were eluted with elution buffer (50 mM Tris/250 mM imidazole, pH 7.5, 300 mM NaCl, 10 mM β-mercaptoethanol).

Human anxA2 bearing the correct N-terminal acetylation was recombinantly expressed and purified as described previously^37^. In brief, *E. coli* DH5α cells expressing anxA2 were resuspended in lysis buffer (50 mM Tris-HCl, pH 8.5; 300 mM NaCl, 10 mM MgCl_2_, 2 mM EGTA and complete protease inhibitor cocktail) and passed three times through a French press at 1,000 psi. After 60 min centrifugation at 40,000 × g supernatants were dialyzed against DE buffer (10 mM Tris-HCl, pH 8.5, 10 mM NaCl, 1 mM EGTA) and loaded onto DEAE columns. The flow through and one additional washing step were dialysed against CM buffer (20 mM sodium acetate pH 5.6, 10 mM NaCl, 1 mM EGTA) and applied to a CM52 column. After extensive washing, anxA2 was eluted between 300 and 500 mM NaCl of a salt gradient. The eluted fractions were dialysed against the appropriate buffers for further experiments. The anxA2–S100A10 tetramer was isolated from porcine small intestine (obtained from a local slaughterhouse) as described before^38^.

### GST pull-down assays

Equal amounts of matrix-bound GST-fusion proteins (50 μg) were equilibrated with IP buffer (20 mM Tris-HCl, pH 7.5, 25 mM NaCl, 10 mM NaF, 1 mM EDTA, 0.1% Triton-X 100) and incubated with either purified protein (10 μg) or with melanoma cell lysate (500 μl, equivalent to ~2 × 10^7^ cells) for 2 hours at 4 °C. To obtain UKRV-Mel-4 lysates, cells were lysed in IP buffer supplemented with 1% Triton-X 100 and Roche complete protease inhibitor cocktail for 60 min at 4 °C, and cleared by centrifugation for 30 min at 400 x g and 4 °C. After extensive washing in IP buffer proteins bound to the matrix were eluted with 2x SDS sample buffer for 5 min at 95 °C. For western blot analysis of the samples, equal protein amounts were separated by 15% SDS-PAGE and transferred onto 0.2 μm nitrocellulose membranes. Rabbit anti-GPR2/CCR10 antibody was from Abcam. The mouse monoclonal anti S100A10 antibody H21, and the mouse monoclonal anti annexin A2 antibody HH7 (MPI Göttingen) were described previously^39^,^40^.

### Cell culture, and RNA interference

The malignant melanoma cell line UKRV-Mel-4^41^,^42^ was maintained in RPMI1640 medium supplemented with 2 mM L-glutamine, 10% Fetal Bovine Serum (Sigma), 100 U ml^−1^ penicillin, 100 μg ml^−1^ streptomycin and 1% non-essential amino acids at 37 °C and 5% CO_2_. For siRNA mediated knockdown of S100A10, cells were transfected with siGENOME SMARTpool human S100A10 siRNA (Dharmacon, M-011766-01) using the Oligofectamine transfection reagent (Invitrogen) according to the manufacturer’s instructions. Non-targeting siRNA (AllStars Negative Control siRNA, Qiagen) was used as negative transfection control in all experiments. Following transfection, cells were cultured for 72 h. To identify siRNA transfected cells for flow cytometric analysis, siRNA was mixed in a 4:1 molar ratio with siGLO Green transfection indicator (Invitrogen) prior to transfection. CCR10 levels and siRNA knockdown efficiencies were analysed by western blotting using the using Odyssey LI-COR infrared imaging system (LI-COR).

### Surface plasmon resonance (SPR) measurements

Measurements were performed using a Biacore 3000 instrument (GE Healthcare). GST and GST-CCR10 CT were immobilized on a CM5 chip using the Amine Coupling Kit (GE Healthcare) following the manufacturer’s instructions. Briefly, the surface of a blank CM5 chip was activated by a 7 min injection of 0.2 M 1-ethyl-3-(3-dimethylaminopropyl)-carbodiimide (EDC) and 0.05 M N-hydroxysuccimide (NHS) in a 1:1 ratio at a flow rate of 5 μl min^−1^. GST or GST-CCR10 CT at 30 μg ml^−1^ in 10 mM sodium acetate, pH 4.5 was immobilized to a level of 500–2,500 resonance units (RU) using manual injections. The surface was deactivated with a subsequent 7 min injection of 1 M ethanolamine-HCl, pH 8.5. HBS-P buffer (10 mM Hepes, pH 7.4, 150 mM NaCl, 0.005% v/v Tween-20) was filtered through 0.22 μM filters (Millipore), degassed and used as running buffer at a flow rate of 5 μl min^−1^ for immobilization.

For interaction analysis, six concentrations of each HIS-tagged S100A10 derivative were injected, starting with the lowest concentration at a flow rate of 30 μl min^−1^ for 3 min (association) and followed by a 9 min dissociation step with running buffer only. After each cycle, the surface was regenerated with a 5 μl pulse injection of 50 mM NaOH. Background binding to GST alone was subtracted, and HBS-P injection was used as blank. Data were recorded as sensorgrams (response units [RU] versus time) at 25 °C with the BIACORE 3000 control software Version 3.2. Data were fitted to a single binding site model, and the dissociation constant (*K*_D_) was calculated according to the Scatchard equation using the BIAevaluation software Version 4.1. Each titration series was repeated multiple times leading to at least six data points per S100A10 derivative and concentration.

### Flow cytometric analysis of CCR10 cell surface presentation

Cells were split and one aliquot was stained with either Phycoerythrin (PE) conjugated rat monoclonal anti-human CCR10 or isotype-matched antibodies (FAB3478P, IC006P, R&D Systems) for 45 min on ice. Samples were analysed employing a FACSCalibur and CellQuest software. Viable cells were gated by using forward and side scatter characteristics. To compare CCR10 cell surface presentation in S100A10 ablated cells and cells transfected with control siRNA, geometric mean fluorescence intensities (MFI) of siRNA-transfected cells (detected via emission of siGLO green transfection indicator) were measured. Non-specific binding determined in the isotype-treated samples was subtracted from all values.

### Microscopic Analysis

For immunofluorescence staining, cells were fixed for 5 min with ice cold methanol, additionally treated with 4% (w/v) paraformaldehyde for 10 min, blocked with 2% BSA (Carl Roth) in PBS, and incubated with the appropriate first (Sigma) and secondary (Invitrogen) antibodies. Nuclei were stained with DRAQ5. Cells were mounted in Mowiol 4-88 supplemented with 1% N-propyl gallate.

Confocal microscopy was carried out using a LSM 780 META microscope (Carl Zeiss) equipped with a Plan-Apochromat 63x/1.4 oil immersion objective. Total internal reflection fluorescence (TIRF) microscopy employed an Olympus IX71 TIRF Microscope (Olympus) and the MetaMorph software (Molecular Devices). Epifluorescence and TIRF images were acquired using the same exposure settings. S100A10 ablated cells were identified by epifluorescence. Quantitative evaluation of CCR10 mean gray values (TIRF field at 488 nm) of 162 cells transfected with non-targeting control siRNA and 144 cells transfected with S100A10 specific siRNA was performed using the ImageJ software (V 1.49a, National Institute of Health, USA). Mean values of empty areas were subtracted.

### Proximity Ligation Assay (PLA)

UKRV-Mel-4 cells were fixed and labelled with primary antibodies against CCR10 (Abcam) and β-actin (Sigma), with the S100A10 antibody H21^39^, or the anxA2 antibody HH7^40^. Subsequently, PLA was performed using Duolink® II *In Situ* PLA (Olink) according to manufacturer’s instructions. Samples were analysed for DAPI and PLA signals by confocal microscopy. For quantitative analysis, maximum intensity projections of stacks (each consisting of 10–12 confocal images along the z-axis) were computed using the ZEN software (Black Edition 2011, Carl Zeiss). Nuclei and PLA-spots were counted using the “Cell Counter” plugin of the ImageJ software.

## Additional Information

**How to cite this article**: Hessner, F. *et al.* CC chemokine receptor 10 cell surface presentation in melanocytes is regulated by the novel interaction partner S100A10. *Sci. Rep.* **6**, 22649; doi: 10.1038/srep22649 (2016).

## Supplementary Material

Supplementary Information
