# Supplementary material for: CC chemokine receptor 10 cell surface presentation in melanocytes is regulated by the novel interaction partner S100A10
Source: Sci Rep. 2016 Mar 4;6:22649. doi: 10.1038/srep22649 (PMC4778132; doi:10.1038/srep22649)
Supplement: Supplementary Information [file srep22649-s1.pdf]

Supplementary Information

CC chemokine receptor 10 cell surface presentation in melanocytes is regulated by the novel interaction partner S100A10

F Hessner, C P Dlugos, T Chehab, C Schaefer, B Homey, V Gerke, T Weide, H Pavenstädt, U Rescher

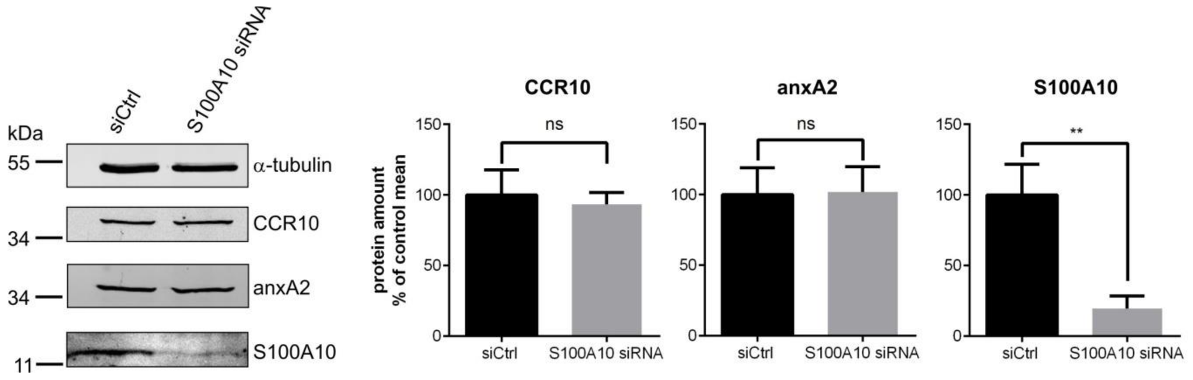

Supplementary Figure S1. S100A10 depletion does not alter CCR10 expression levels. Lysates of UKRV-Mel-4 cells transfected with non-targeting control siRNA (siCtrl) or S100A10-targeting siRNA were immunoblotted for anxA2 and S100A10 to confirm the successful knockdown, CCR10 to determine CCR10 expression levels, and tubulin as a loading control (left). For quantitative analysis of western blots, a mean value of the respective band intensities of five independent transfection experiments was calculated for the control samples. The band intensities of the control as well as the test samples were then expressed as percentage of the respective control mean. Significance of means ± s.e.m. from five independent experiments were statistically analysed by two-sided unpaired *t*-test (right). A *p*-value of < 0.05 was considered significant. ** $p < 0.01$; ^{ns} not significant.

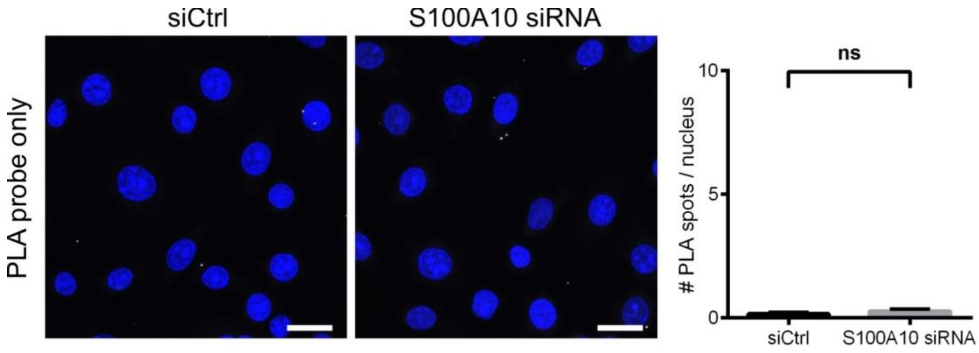

Supplementary Figure S2. Negative control of PLA probes without primary antibody. Nuclei are stained with DAPI. Samples were analysed using two-sided unpaired t-test. Scale bars represent 20 µm. ^{ns} not significant.

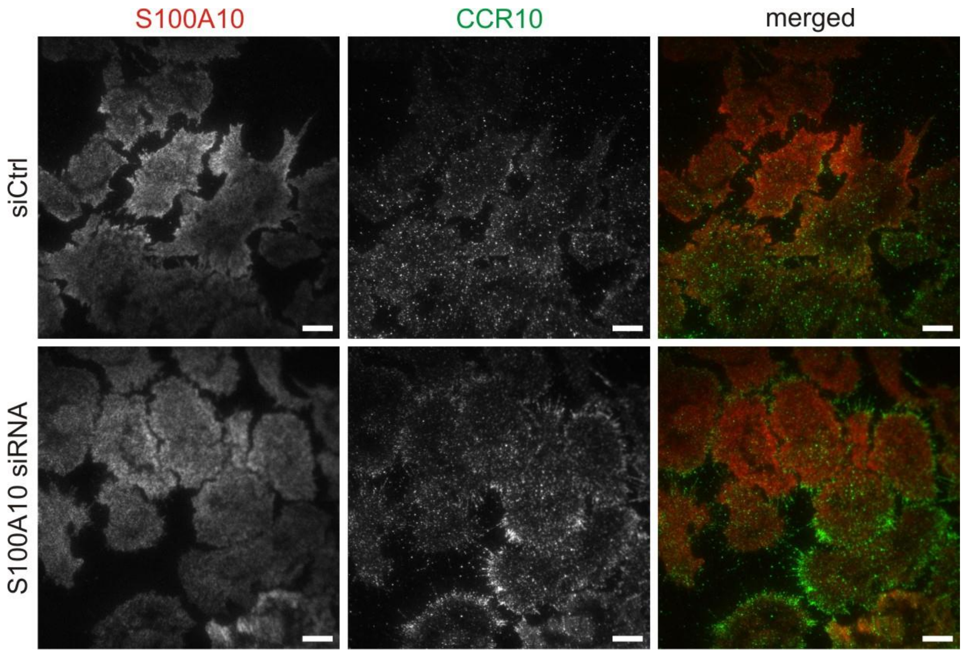

Supplementary Figure S3. TIRF images of CCR10 and S100A10 distribution in UKRV-Mel-4 cells. Note that no apparent colocalisation of the two molecules is observed at sites of increased CCR10 staining. Nuclei were stained with DAPI, scale bars represent 20 µm.

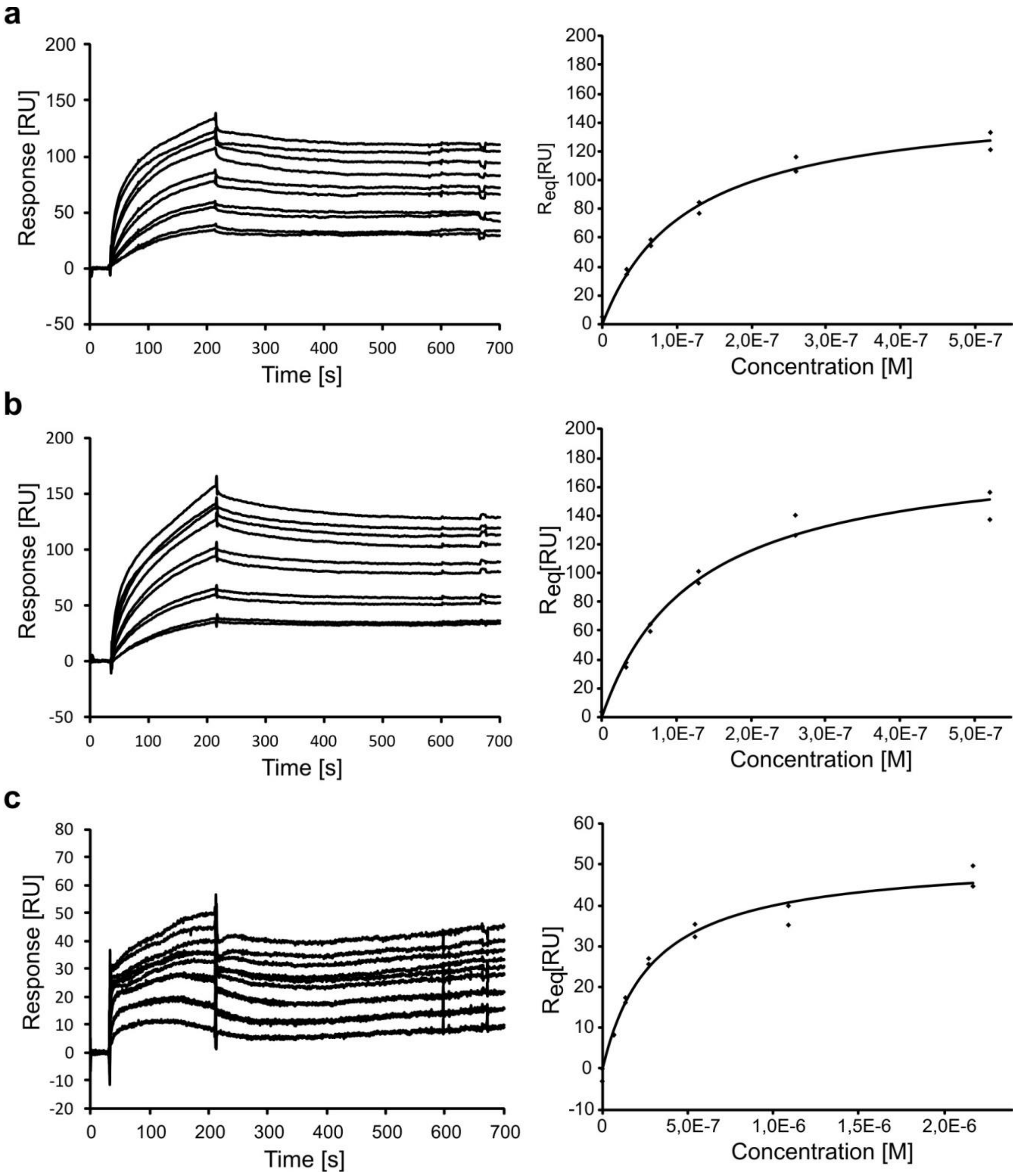

Supplementary Figure S4. SPR interaction analysis of CCR10 and S100A10 derivatives impaired in annexin A2 binding. GST-CCR10 CT was immobilized on a CM5 chip and (a) HIS-tagged S100A10 C82S, (b) C82Q and (c) 85 Stop mutant proteins were injected as concentration series for kinetic analysis. Response of single injections (without buffer only control) are shown on the left and calculated steady state kinetics on the right. RU = resonance units, R_{eq} = response at equilibrium.

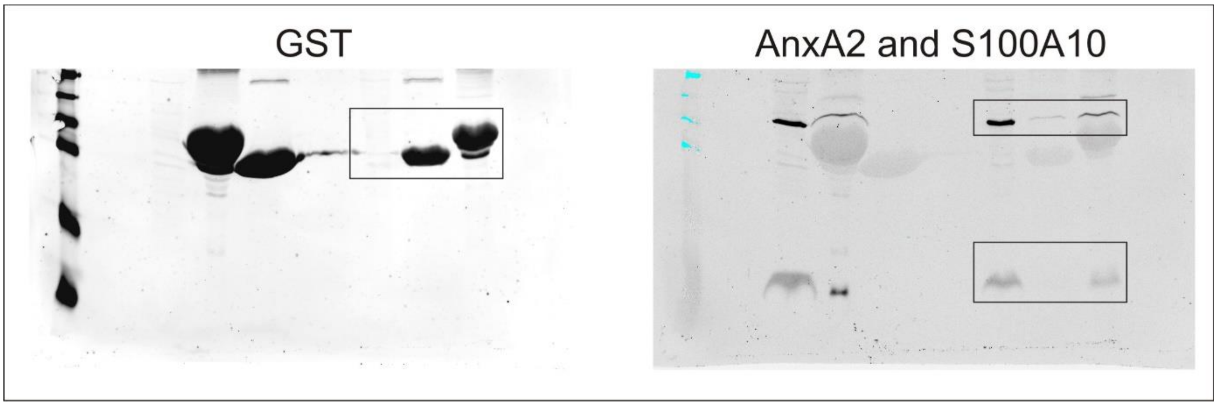

Fig.3a

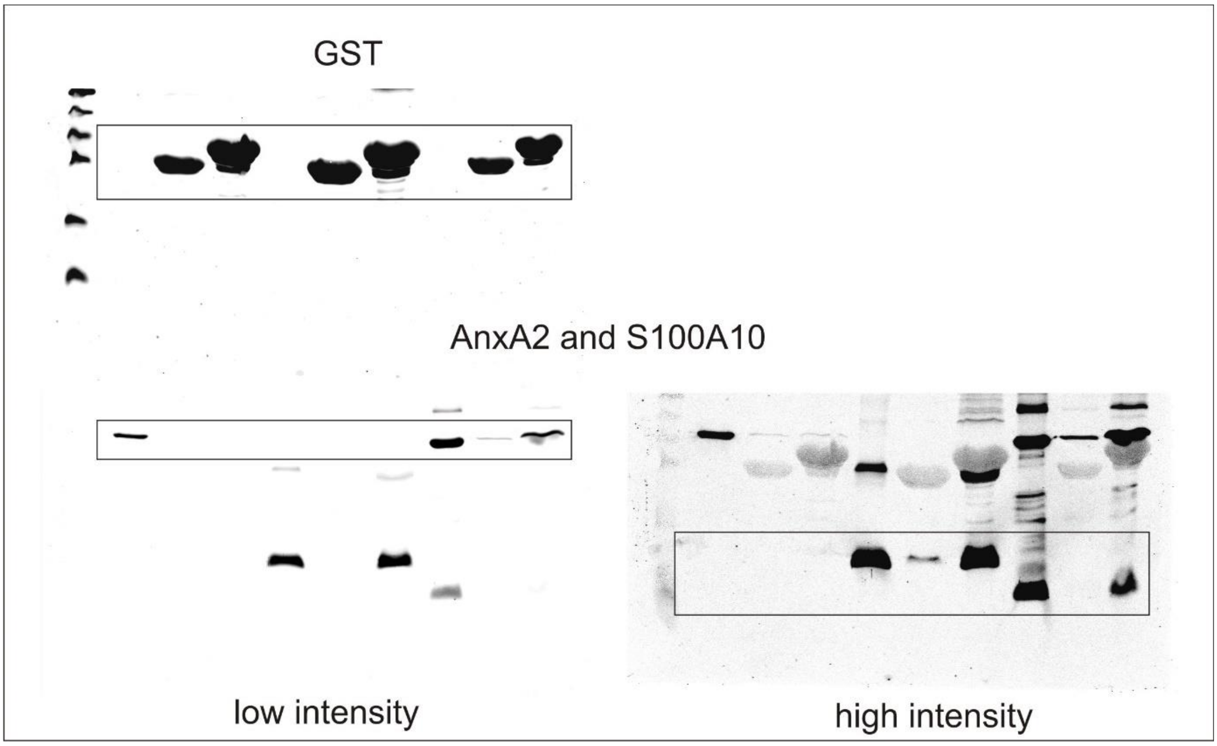

Fig.3b

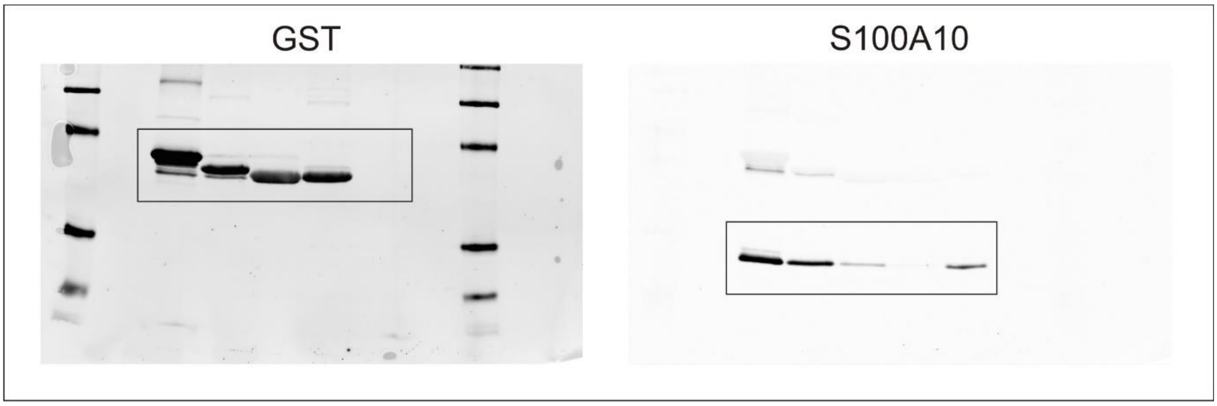

Fig.4b

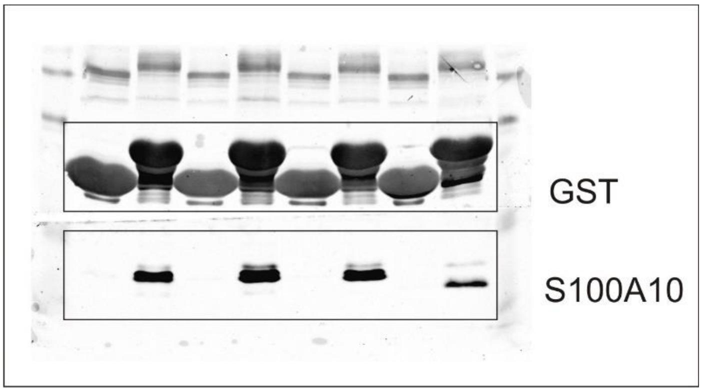

Fig. 5b

Supplementary Figure S5. Scans of uncropped blots. Boxed regions indicate cropped regions. Note that for optimal detection of AnxA2 and S100A10, the same blot was scanned at different intensity settings (Fig. 3b).
